# Preparation and Evaluation of Metronidazole-Loaded Pectin Films for Potentially Targeting a Microbial Infection Associated with Periodontal Disease

**DOI:** 10.3390/polym10091021

**Published:** 2018-09-13

**Authors:** Taepin Junmahasathien, Pattaraporn Panraksa, Paytaai Protiarn, Doosadee Hormdee, Rajda Noisombut, Nutthapong Kantrong, Pensak Jantrawut

**Affiliations:** 1 Department of Pharmaceutical Sciences, Faculty of Pharmacy, Chiang Mai University, Chiang Mai 50200, Thailand; phoenixj035@hotmail.com (T.J.); pattaraporn.prs@gmail.com (P.P.); pt.protiarn@gmail.com (P.P.); 2 Department of Periodontics, Faculty of Dentistry, Khon Kaen University, Khon Kaen 40002, Thailand; nootdoosadee@hotmail.com; 3 Department of Community Dentistry, Faculty of Dentistry, Khon Kaen University, Khon Kaen 40002, Thailand; rajnoi@kku.ac.th; 4 Department of Restorative Dentistry, Faculty of Dentistry, Khon Kaen University, Khon Kaen 40002, Thailand

**Keywords:** pectin, film, metronidazole, antimicrobial activity, periodontal disease

## Abstract

The objective of this study was to develop the metronidazole loaded high and low methoxyl pectin films (HM-G-MZ and LM-G-MZ) for the treatment of periodontal disease. The films were prepared by pectin 3% *w*/*v*, glycerin 40% *w*/*v*, and metronidazole 5% *w*/*v.* The developed films were characterized by scanning electron microscope and evaluated for thickness, weight variation, and elasticity. The developed films showing optimal mechanical properties were selected to evaluate radial swelling properties, in vitro release of metronidazole and the antimicrobial activity against *Porphyromonas gingivalis* and *Aggregatibacter actinomycetemcomitans* by the disc diffusion method. The results demonstrated that LM-MZ and HM-G-MZ films were colorless and yellowish color, respectively, with the film thickness around 0.36–0.38 mm. Furthermore, both films exhibited good elasticity with low puncture strength (1.63 ± 0.37 and 0.84 ± 0.03 N/mm^2^, respectively) and also showed slight increase in radial swelling, so that they could be easily inserted and fitted into the periodontal pocket during a clinical use. However, HM-G-MZ showed a decrease in radial swelling after 1 h due to the film erosion. The in vitro release study of LM-G-MZ showed a burst release that was initially followed by a slow release rate profile, capable to maintain the therapeutic level in periodontal pocket for seven days, whereas HM-G-MZ showed an immediate release profile. The cumulative percentage of metronidazole release from HM-G-MZ was less than LM-G-MZ during the first 5 min as metronidazole was in a crystalline form inside HM-G-MZ film. For antimicrobial activity test, both films showed the inhibitory effect against *P. gingivalis* and *A. actinomycetemcomitans*, and there was no difference in the inhibition zone between LM-G-MZ and HM-G-MZ. The present study showed, for the first time, that low methoxyl pectin film containing glycerin and metronidazole could be potentially considered as a promising clinical tool for the drug delivery via intra-periodontal pocket to target an oral disease that is associated with polymicrobial infection.

## 1. Introduction

Periodontal disease is one of the most prevalent oral diseases affecting more than 30% of Thai community [1]. It is an inflammatory disease of bacterial origin that affects the tooth-supporting tissues [2]. The appearance of periodontal pockets is the first clinical manifestation of periodontal diseases that offers a favorable niche with low oxygen tension for bacterial colonization [3]. Thus, the treatment of periodontitis mainly focuses on the reduction of the total bacterial, which is the primary cause of periodontal diseases [4]. Conventional treatment method by oral administration of antimicrobial agents must be given in high doses to maintain the effective concentration in gingival crevicular fluid. However, high doses of antimicrobials cause side effects, such as gastrointestinal disorders, development of resistant bacteria, and suprainfection. Thus, the drug delivery system of antimicrobial agents via intra-periodontal pockets has been invented for the possibility to overcome the clinical challenge that is encountered during the systemic administration of antimicrobials [4].

Since periodontal disease is associated with polymicrobial infection, antibacterial treatment such as metronidazole might be useful for improving the condition of the inflamed tissues. Metronidazole is a partially hydrophilic drug, which effectively inhibits an infection of anaerobic microorganisms and protozoa [5,6]. It is used to treat the infections that are caused by anaerobic bacteria, including intra-abdominal infections, skin and skin structure infections, gynecological infections, bacterial septicemia, bone and joint infections, central nervous system infections, lower respiratory tract infections, endocarditis, and also periodontal disease that caused by *Gardnerella vaginalis* [7,8,9].

However, there are still limited approaches used for preparing the formulations of sustained/controlled-release metronidazole films aided in an intra periodontal pocket application. In 2009, metronidazole-loaded bioabsorbable films consisting of different ratios of poly(dl-lactic acid) (PDLLA) and poly(dl-lactide-co-glycolide) (PDLGA) was developed. The films demonstrated the controlled-release profile of metronidazole lasting longer than one-month for treating periodontal pocket infections [10]. In addition, metronidazole and amoxicillin were also loaded in PDLGA and PDLLA films in another study [11]. Drug-release study showed that during the first 16 days, the released quantities of drugs were higher than the minimum inhibitory concentration (MIC) needed against the growth of various organisms causing periodontal disease. The aim of the present study was to prepare metronidazole-loaded film while using high (HM) and low methoxyl pectin (LM) as polymers for periodontal pocket application. The prepared formulations were evaluated to ensure optimum film characteristics, an antibacterial efficiency related to its potential clinical use.

## 2. Materials and Methods

### 2.1. Materials

Non-amidated low methoxy pectin (Unipectine; DE = 29%) was purchased from Cargill^TM^, Saint Germain, France. High methoxyl pectin (DE = 60%) was purchased from Du Pont^®^, Wilmington, DE, USA. Metronidazole was purchased from Alfa Aesar, Tewksbury, MA, USA. Calcium chloride (CaCl_2_) and disodium hydrogen phosphate were purchased from Merck (Damstadt, Germany). Distilled water served as the solvent for preparing film solutions. All of the reagents were analytical grade.

### 2.2. Microorganisms

Two different species of periodontal pathogen were utilized for testing the ability of metronidazole-containing films to statically inhibit bacterial growth, as well as to kill periodontal microbes. *Porphyromonas gingivalis* strain ATCC 33277 and *Aggregatibacter actinomycetemcomitans* strain ATCC 43718 were obtained from the stock collection of the Oral Biology Research Division, Khon Kaen University. Both bacteria were grown in Wilkins-Chalgren Anaerobe broth (Oxoid, Hampshire, England) consisting of tryptone (10 g/L), yeast extract (5 g/L), menadione (0.5 mg/L), and hemin (5 mg/L). A culture of *P. gingivalis* was incubated in a 3 mL broth overnight in a 37 °C anaerobic condition that was achieved by using AnaeroPack^®^-Anaero (Mitsubishi Gas Chemical, Tokyo, Japan) in combination with a leak-proof jar, otherwise maintained on blood agar plates. *A. actinomycetemcomitans* was grown in a similar broth but under a CO_2_ condition.

### 2.3. Preparation of Metronidazole-Loaded Pectin Film

HM or LM pectin films containing metronidazole were prepared while using conventional casting or modified ionotropic gelation techniques, respectively. Sample code and film compositions were shown in Table 1. For metronidazole-loaded HM film, HM solution was prepared by dispersing 3% *w*/*v* of HM in distilled water at 60 °C for 1 h and allowed for complete hydration and swelling overnight, after which 5% metronidazole together with or without glycerin 40% *w*/*v* of polymer was added to HM solution, gently stirred using a glass rod, and left until all of the air bubbles disappeared and casted in a horizontally leveled plastic plate. For LM, film formulations comprising of 3% *w*/*v* LM with or without glycerin 40% *w*/*v* and metronidazole 5% *w*/*v* were prepared and casted on a 6 cm × 6 cm dialysis membrane (Cellu-Sep T3/Nominal MWCO: 12,000–14,000 Da, Membrane Filtration Product, Inc., Texas, Seguin, TX, USA). Then, the casted-film forming solution on the membranes were placed on crosslinking agent (3% *w*/*v* CaCl_2_) supported by plastic box for 2 h [12]. The fresh films were subsequently placed on another plastic plates. All of the films were dried in an oven (30 ± 2 °C) for 48 h and carefully removed, inspected for any imperfections, and stored in a desiccator prior to the use.

### 2.4. Film Characterizations

#### 2.4.1. Morphological Characterization of the Films

The morphology was examined while using scanning electron microscopy (SEM) with a JEOL scanning electron microscope (JSM-5410LV, JEOL Ltd., Peabody, MA, USA) at 10 kV under low vacuum mode. The film characterizations were performed without any coating solution at magnifications of ×150. The thickness and surface of films were evaluated.

#### 2.4.2. Film Thickness and Weight Variation

The thickness of each 3 cm × 3 cm sized film was measured at 10 points while using a thickness gauge (GT-313-A, Gotech Testing Machines Inc., Taichung, Taiwan). Ten films in circle shape with 4 mm diameter were individually weighed by analytical balance (PA214, Ohaus Parsippany, NJ, USA). The mean film thickness (in mm) and weight (in mg) with the standard deviation were calculated. 

#### 2.4.3. Mechanical Strength Test

Mechanical strength of the films was tested using a texture analyzer TX.TA plus (Stable Micro Systems, Surrey, UK). An individual sample holder has been constructed to facilitate measurements of a 3 cm × 3 cm sized film samples. Film was fixed by screws between two plates with a cylindrical hole of 9.0 mm diameter (area of the sample holder hole = 63.56 mm^2^). Needle stainless probe (2 mm) was used (probe contact area = 3.14 mm^2^). The texture analyzer was adjusted for the probe’s forward movement at a velocity of 1.0 mm/s. Measurement started when the probe had contacted the sample surface (triggering force). The probe moved on at constant speed until the film was torn. The applied force and displacement (penetration depth) were recorded. All of the experiments were conducted at a room condition (25 °C, 70% relative humidity). The mechanical strength of the film was characterized by maximum applied force (Max. force), displacement, area under the force-displacement curve (AUG), and puncture strength [13], and reported as the average value from the obtained results. The film formulations that showed superior properties that are associated with mechanical strength were selected for further experiments.

#### 2.4.4. Swelling Study 

Swelling behavior of metronidazole-loaded pectin films was evaluated by the diameter method [14]. Six film discs of 4 mm diameter were allowed to swell on the surface of a custom-made agar plate and were kept in an incubator maintained at 37 °C. The diameter of the swollen discs was measured using a micrometer at predetermined time intervals of 5 min to 1 h for HM-G-MZ and 7 days for LM-G-MZ. The radial swelling was calculated from the following equation: %*S*_d_ *= (D*_t_ − *D*_0_*)/D*_0_. Where %*S*_d_ is the percentage swelling that is obtained by the diameter method, *D*_t_ is the diameter of the swollen disc after time *t*, and *D*_0_ is the original dry disc diameter.

### 2.5. Metronidazole Content

The amount of metronidazole loaded into the selected film (LM-G-MZ and HM-G-MZ) was determined by adding a 4 mm diameter film containing 146.7 µg (11.66 µg/mm^2^) of metronidazole into 2 mL of phosphate buffer, (pH 6.6) under a constant stirring at 400 rpm for 4 h until complete disintegration. The dispersion was filtered and the absorbance measured at 318.5 nm by UV spectroscopy (V530, Jusco, Easton, MD, USA). Metronidazole content was determined from the standard curve of metronidazole in buffer, which was linear with a high correlation coefficient (r^2^ = 0.9994). The following regression equation was obtained: *y =* 0.0541*x +* 0.0138, where *y* is the absorbance and *x* is the concentration of metronidazole (µg/L). The experiment was done in triplicate. The percentages of drug content were calculated.

### 2.6. In Vitro Metronidazole Release Study

Metronidazole-loaded pectin films with a diameter of 4 mm were immersed in 2 mL Tris buffer gingival fluid pH 6.6 at 37 °C up to seven days in semi static conditions to determine the kinetics of metronidazole release from the pectin films. Sodium azide (0.05% *w*/*v w*/*v*) was added to prevent contamination by various microorganisms. At the different time intervals, 0.5 mL samples were collected at 5, 10, 15, 30, and 60 min for HM-G-MZ and until seven days for LM-G-MZ. The samples were diluted 20 times before measured at 318.5 nm by UV spectroscopy. All of the dissolution runs were performed in triplicate.

### 2.7. Preparation of Bacteria for Agar Diffusion Test and Anti-Bacterial Activity of Metronidazole-Loaded Pectin Film

*P*. *gingivalis* and *A*. *actinomycetemcomitans* were grown in 3 mL TYHK broth overnight at 37 °C under anaerobic and CO_2_ condition, respectively. Bacterial cells were prepared by measuring optical density (OD) at 600 nm wavelength and further diluting the cultures to obtain the OD of 0.1 by using sterile TYKH broth. A 100 µL prepared bacterial culture was spread on top of the blood agar plate prior to placing pectin films containing metronidazole. To ensure the killing ability of metronidazole against two tested periodontal pathogens, we also performed the experiment to determine the minimum inhibitory concentration (MIC) of metronidazole and incorporated on the blood agar plates. Briefly, two-fold dilution of metronidazole was made using ultrapure water to achieve the dose range of 0.16 to 5 mg/mL. A 10 µL metronidazole at each concentration was dropped on the sterile 6 mm disc separately. In addition, we used the disc containing 10 µL ultrapure water as a negative control. Metronidazole-loaded pectin films were subsequently placed on the blood agar inoculated with each bacterial strain. Our test samples were incubated under the optimal conditions for bacterial growth for 48 h until a lawn of bacterial colonies was clearly seen. The diameters of an inhibition zone in which the width of the film was included were measured while using Mitutoyo^®^ Digimatic caliper (Mitutoyo Corporation, Kanagawa, Japan). Three independent experiments were performed in triplicate.

### 2.8. Statistical Analysis 

All data are presented as mean ± SD. One-way ANOVA was used to evaluate the significance of differences at *p*-value < 0.05, unless otherwise stated. Statistical analysis was performed while using SPSS software version 16.0 (SPSS Inc., Chicago, IL, USA)

## 3. Results and Discussion

### 3.1. Film Preparation and Characterization

**Figure 1:**
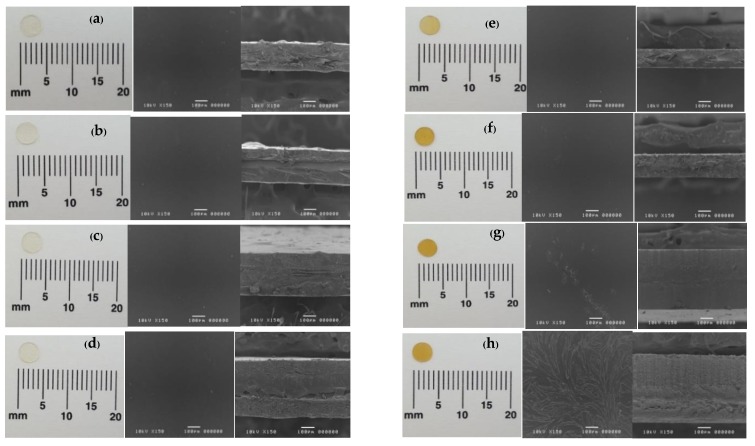
Morphology (left) and scanning electron microscopy of the surface (middle) and thickness (right) at 150× magnifications of LM (**a**)**,** LM-G (**b**), LM-MZ (**c**), LM-G-MZ (**d**), HM (**e**), HM-G (**f**), HM-MZ (**g**), and HM-G-MZ (**h**), LM: Low methoxyl pectin film; G: Glycerin; MZ: Metronidazole; HM: high methoxyl pectin film.

All of the developed films were transparent and odorless. All types of LM films were slightly yellowish to colorless, while HM films provided yellowish to light brown color (Figure 1). Generally, pectin extracted from apple pomace and citrus peel was high methoxyl pectin, which has yellowish to light brown powder color. Whereas, a low methoxyl pectin, which is a commercially available form, is obtained by de-esterification of HM pectin has faded color [15]. From Figure 1, loading glycerin into film was not affected to the appearance and morphology of the films. Additionally, glycerin as a plasticizer also provided a flexible with less brittle film when compared to non-glycerin film. When loaded metronidazole into films, it caused less transparency than unloaded films. SEM images showed the crystalline form of metronidazole in HM-MZ and HM-G-MZ films (Figure 1g,h). This might be attributed to the method of film preparation resulting in metronidazole recrystallization. During the film preparation, HM remained in solution while LM was a gel-like fluid during drying process. The gel-like fluid was steric structure that retarded the diffusion of the molecule of metronidazole and inhibited crystal growth [16]. Furthermore, metronidazole was crystallized in HM-G-MZ film (Figure 1h) more than that found in HM-MZ film (Figure 1g). With this regard, the addition of glycerin in the HM-G-MZ film possibly generated less water than those of the HM-MZ film. In addition, the hydrophilic property of glycerin contributes to the water hydration phenomenon surrounding metronidazole molecule. This leads to supersaturation and subsequent crystal formation of metronidazole [17]. Moreover, plasticizer, such as glycerin, could modify the organization of film structure and decreased attractive intermolecular forces. These might lead to the increase of free volume and chain mobility, which allowed for the ease of crystal growth [18]. 

Determination of film thickness and weight variation was performed to ensure the consistency of film preparation as well as the amount of the drug in the film. The results showed that the comparison between different type of custom-made films (*n* = 10 per group) demonstrated a slight difference of thickness and weight (Table 2). Notably, the film thickness was increased when metronidazole and/or glycerin was loaded into the films. The increased film thickness as a result of the addition of plasticizer is in agreement with an earlier study [12]. The thickness of LM-G-MZ and HM-G-MZ film were optimized to be approximately 0.37 mm, which would be appropriate for inserting into periodontal pocket with the width less than 0.5–3.0 mm [19]. For drug loading contents of metronidazole films, the contents were 101 ± 3.44 and 100 ± 1.50% in LM-G-MZ and HM-G-MZ films, respectively. The drug loading result indicated that metronidazole powder was dissolved and incorporated directly into polymer solution, still stable during the film preparation process, and also exhibited the homogenously blended metronidazole in both films. Thus, both LM-G-MZ and HM-G-MZ demonstrated the uniformity with respect to metronidazole content.

### 3.2. Mechanical Properties 

The result of mechanical strength of metronidazole pectin films was shown in Table 3. Technically, maximum force, area under the force-displacement curve (AUC), puncture strength, and high displacement (penetration depth) values are associated with the elasticity of the film [13]. In this study, LM and HM films with glycerin showed significantly lower maximum force and puncture strength than those films without glycerin (*p* < 0.05). The effect of glycerin on the properties of pectin film was similar to another study demonstrating that, even though the addition of glycerin leads to the reduction of a static modulus, dynamic modulus, and tensile strength, the elongation of the films was found to be increased [20]. Adding metronidazole in non-glycerin film apparently increased the mechanical properties especially of LM-MZ showing that the puncture strength was increased from 3.26 to 4.48 N/mm^2^ but not significantly increased in HM-MZ. While, adding glycerin in metronidazole-loaded film showed a significant decrease in the mechanical properties (*p* < 0.05). For example, the puncture strengths of LM-G and LM-G-MZ were 2.05 and 1.63 N/mm^2^, whereas puncture strengths were 1.37 and 0.84 N/mm^2^ for HM-G and HM-G-MZ, respectively. Interestingly, the reduction of mechanical strengths in high methoxyl pectin containing metronidazole films (HM-MZ and HM-G-MZ) was higher than those that were comprised of low methoxyl pectin (LM-MZ and LM-G-MZ). This discrepancy might be contributed by the film network and the difference of crystal state of metronidazole in LM and HM. This result is consistent with a previous study of Preis et al. reporting that amorphous state provided higher mechanical strength. Due to the strong interaction of molecules, rigidity and large size of metronidazole, crystal state provided steric effect as a barrier between the interaction of polymer chain. While in amorphous state, the interaction between drug molecules was lower than crystalline form, known to be less affected by the interaction between polymer chain [13].

One crucial factor of film formulations is the mechanical strength of the films during the development and production process. In this study, metronidazole films without glycerin (LM-MZ and HM-MZ) were very brittle and easily broken when cut into small circular shape prior to use in further experiments. Therefore, HM-G-MZ and LM-G-MZ films were chosen, since they provided superior mechanical properties. 

### 3.3. Film Swelling Behavior

**Figure 2:**
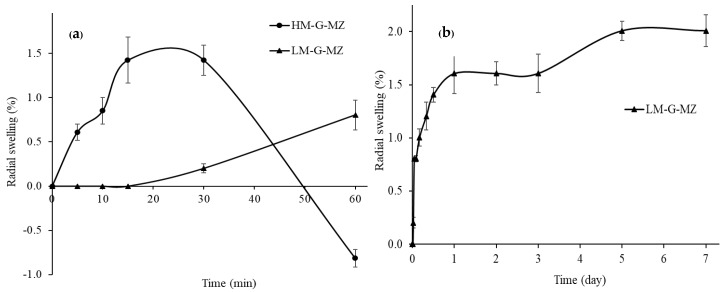
The swelling behavior of LM-G-MZ and HM-G-MZ films (*n =* 6) on agar plate by diameter method at different time intervals from 0 to 1 h for both films (**a**) and up to seven days (**b**) for LM-G-MZ film.

LM-G-MZ film slightly swelled 0.8% within 60 min (Figure 2a), maintained stable, and swelled with the maximum swelling of 1.81% from five to seven days (Figure 2b). The small degree of swelling may be suitable when the film is inserted via periodontal pocket to achieve maximum retention. For high methoxyl pectin, HM-G-MZ film dramatically swelled to the maximum swelling of 1.42% in 15 min then the diameter of the disc continuously reduced until smaller than the initial diameter (Figure 1a) and completely dissolved after 1 h. This indicated the erosion of HM-G-MZ film on the agar plate during the swelling experiment. The difference of LM and HM film swelling behavior might be caused by the method of film preparation. LM film was produced by ionotropic gelation techniques. The gelation formed by the presence of calcium ions, was involved with ionic interactions between the cations and the negative charged cavities formed by low methoxyl pectin chains [18]. This three-dimensional structure of LM provided the swelling that could hold a large amount of water molecules into their structure. On the other hand, HM film was constructed by conventional casting method. The network at the junction zones took place through the formation of aggregates with hydrogen bonding and hydrophobic chain interactions [21]. This causes a weak HM-G-MZ film’s structure, as compared to the three-dimensional structure of LM-G-MZ film. These results were also correlated with the lower puncture strength of HM-G-MZ than those of LM-G-MZ films. Thus, when HM-G-MZ film swelled, a large amount of water in the structure led the structural collapse and erosion of film was occurred [22,23].

### 3.4. In Vitro Metronidazole Release 

**Figure 3:**
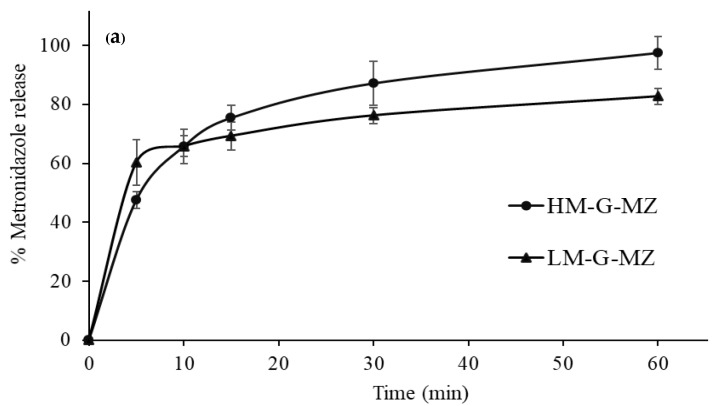

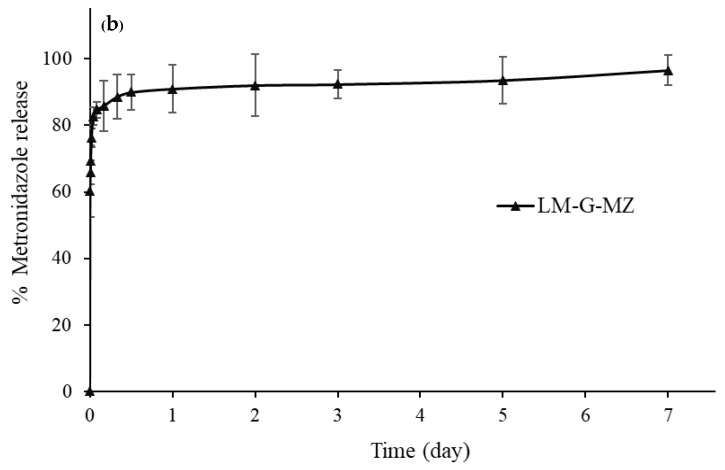
Dissolution profiles of LM and HM films loaded metronidazole (*n =* 3) in Tris buffer pH 6.6 at different time intervals from 0 to 1 h for both films (**a**) and up to seven days (**b**) for LM-G-MZ film.

LM-G-MZ film showed a fast metronidazole release at the initial stage, up to 60.26% within 5 min followed by a slow release rate from 60% to 80% in 60 min (Figure 3a). In addition, LM-G-MZ film showed the capability to sustain a slow metronidazole release profile at approximately 92% release until the day 7 (Figure 3b). This two-phase release profile was also correlated with the swelling behavior of LM-G-MZ. On the other hand, the metronidazole release profile of HM-G-MZ film showed a fast release up to 75.37% within 15 min and reached 97.40% in 1 h. As a result, rapid dissolution was observed in HM-G-MZ by the erosion of high methoxyl pectin. However, the cumulative percentage of HM-G-MZ metronidazole release was less than LM-G-MZ film during the first 5 min. This could be explained by the formation of crystalline form of metronidazole on the HM-G-MZ film surface, which exhibited the slower dissolution rate than the amorphous form on LM-G-MZ film surface [24]. According to two different release profiles, it is recommended that the HM-G film is appropriate for immediate release drug delivery system while LM-G film might be recommended for a prolonged release drug delivery system. In the case of metronidazole for periodontal pocket infections, the LM-G-MZ was suitable possibly due to the prolonged release of drug in periodontal pocket to maintain the therapeutic concentration for up to seven days. This would hence increase the therapeutic efficiency of metronidazole in the periodontal pocket where microbial infection is a primary cause and this invented device might also provide a better compliance from dental patients.

### 3.5. Anti-Bacterial Activity of Metronidazole-Loaded Pectin Film

**Figure 4:**
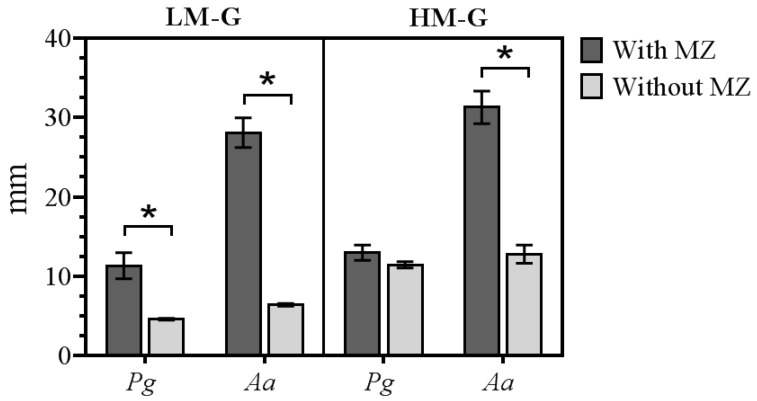
Differential inhibition of bacterial growth illustrated by the inhibition zone. The average diameter ± standard deviation (in mm) of inhibition zones of LM-G and HM-G films loaded metronidazole (*n =* 3) against the growth of *P. gingivalis* (*Pg*) and *A. actinomycetemcomitans* (*Aa*) was shown. LM-G and HM-G films (depicted in grey bars) without metronidazole served as negative controls. Statistical difference was determined by using paired *t*-test to determine the significance, indicated by asterisk (*), at *p* < 0.05.

We first determined the MIC of metronidazole in killing *P*. *gingivalis* and *A*. *actinomycetemcomitans* and found that the growth of *P*. *gingivalis* and *A*. *actinomycetemcomitans* was inhibited at the concentration of 5 mg/mL and 2.5 mg/mL, respectively. This suggests the killing selectivity of metronidazole on different bacterial species. Next, we investigated the efficacy of our LM and HM films in killing two tested bacterial strains. Test films were sterilized by ethylene oxide prior to testing the anti-bacterial activity. The results of the antimicrobial activity of the films against the growth of *P*. *gingivalis* and *A*. *actinomycetemcomitans* for 48 h using agar disk diffusion method are shown in Figure 4. LM-G-MZ and HM-G-MZ film exhibited no significant difference of the inhibition zone, which were 11.34 ± 1.65 and 12.98 ± 0.97 mm for *P. gingivalis*; 28.09 ± 1.88 and 31.28 ± 2.05 mm for *A*. *actinomycetemcomitans*, respectively. The rapid HM-G-MZ film dissolution might be involved in the slightly greater clear zone of HM-G-MZ than LM-G-MZ film, as shown in grey bars of Figure 4. Moreover, the film without metronidazole S (LM-G and HM-G) showed negligible inhibition zone. In fact, glycerin which is an excipient in the film formulation, also have antimicrobial activity on its own [25]. *P*. *gingivalis* and *A*. *actinomycetemcomitans* have been considered to be major periodontal pathogens that are associated with severe and chronic periodontitis [26,27,28]. In the present study, LM-G-MZ film could be exploited as a device possessing a prolonged metronidazole release property for the treatment of periodontal disease by targeting *P. gingivalis* and *A. actinomycetemcomitans.*

## 4. Conclusions

In this study, pectin films that were loaded with metronidazole were prepared and evaluated for their morphology, mechanical properties, loading content, metronidazole release and anti-bacterial activity. Addition of glycerin in pectin film formulations decreased the maximum applied force and increased the displacement, which appeared to improve the film flexibility when compared to the films designed without glycerin. All of the pectin film formulations exhibited fairly uniformed drug content with 100% metronidazole. Rapid dissolution was observed in HM-G-MZ film. However, LM-G-MZ film showed a fast release rate at the initial stage, followed by a slow release rate that is capable of maintaining approximately 90% release up to seven days. According to the test of antimicrobial activity against *P. gingivalis* and *A. actinomycetemcomitans*, the HM-G-MZ and LM-G-MZ films showed similar inhibitory effect. This study indicated that LM pectin film prepared by ionotropic gelation technique in which intramolecular cross-links occurred between negatively charged carboxyl groups of LM and the positively charged of cross-linked solution was suitable for sustained-release drug dosage forms. Whereas, HM pectin film using the solvent casting method might be more appropriate for an immediate-release product. These results could be useful for further study to investigate the mucoadhesive properties, as well as human skin irritation, so as to contribute to further development of these thin films containing antimicrobial agent for eliminating a microbial infection in the periodontal pockets.

## Figures and Tables

**Table 1:** Film compositions.

Sample Code	LM Pectin (%)	HM Pectin (%)	Glycerin (%)	Metronidazole (%)
LM	3	-	-	-
LM-G	3	-	40	-
LM-MZ	3	-	-	5
LM-G-MZ	3	-	40	5
HM	-	3	-	-
HM-G	-	3	40	-
HM-MZ	-	3	-	5
HM-G-MZ	-	3	40	5

**Table 2:** Film thickness and weight variation.

Film	Thickness (mm ± SD)	Weight (mm ± SD)
LM	0.30 ± 0.02 ^a^	4.83 ± 0.25 ^a^
LM-G	0.36 ± 0.01 ^b^	5.50 ± 0.65 ^b^
LM-MZ	0.34 ± 0.02 ^c^	5.40 ± 0.25 ^b^
LM-G-MZ	0.37 ± 0.01 ^b^	6.23 ± 0.18 ^c^
HM	0.25 ± 0.04 ^d^	4.62 ± 0.92 ^a^
HM-G	0.35 ± 0.03 ^c^	6.49 ± 0.61 ^c^
HM-MZ	0.28 ± 0.03 ^a^	4.53 ± 0.41 ^a^
HM-G-MZ	0.37 ± 0.01 ^b^	6.92 ± 0.30 ^d^

For each test, means with the same letter are not significantly different. Thus, means with the different letter, e.g., ‘a’ or ‘b’ are statistically different (*p* < 0.05).

**Table 3:** Mechanical properties of the films.

Film	Max. Force (N)	Displacement (mm)	Puncture Strength (N/mm^2^)	AUC to Peak (N·mm)
HM	12.59 ± 1.19 ^a^	0.74 ± 0.23 ^a^	4.01 ± 0.38 ^a^	3.15 ± 0.80 ^a^
HM-G	4.31 ± 0.58 ^b^	1.21 ± 0.08 ^b^	1.37 ± 0.19 ^b^	2.26 ± 0.29 ^b^
HM-MZ	12.81 ± 1.07 ^a^	0.70 ± 0.03 ^a^	4.08 ± 0.34 ^a^	3.33 ± 0.36 ^a^
HM-G-MZ	2.65 ± 0.08 ^c^	1.15 ± 0.01 ^c^	0.84 ± 0.03 ^c^	1.24 ± 0.08 ^c^
LM	10.24 ± 1.50 ^d^	1.13 ± 0.02 ^c^	3.26 ± 0.48 ^d^	3.63 ± 0.49 ^a^
LM-G	6.44 ± 0.09 ^b^	1.02 ± 0.05 ^d^	2.05 ± 0.03 ^e^	2.24 ± 0.14 ^b^
LM-MZ	14.09 ± 0.69 ^e^	1.16 ± 0.08 ^c^	4.48 ± 0.22 ^a^	4.84 ± 0.41 ^d^
LM-G-MZ	5.13 ± 1.16 ^b^	1.06 ± 0.08 ^d^	1.63 ± 0.37 ^b^	2.08 ± 0.25 ^b^

For each test, means with the same letter are not significantly different. Thus, means with the different letter, e.g., ‘a’ or ‘b’ are statistically different (*p* < 0.05).
